# Assessment of T-cell Reactivity to the SARS-CoV-2 Omicron Variant by Immunized Individuals

**DOI:** 10.1001/jamanetworkopen.2022.10871

**Published:** 2022-04-22

**Authors:** Lorenzo De Marco, Silvia D’Orso, Marta Pirronello, Alice Verdiani, Andrea Termine, Carlo Fabrizio, Alessia Capone, Andrea Sabatini, Gisella Guerrera, Roberta Placido, Manolo Sambucci, Daniela F. Angelini, Flavia Giannessi, Mario Picozza, Carlo Caltagirone, Antonino Salvia, Elisabetta Volpe, Maria Pia Balice, Angelo Rossini, Olaf Rötzschke, Emiliano Giardina, Luca Battistini, Giovanna Borsellino

**Affiliations:** 1 Neuroimmunology Unit, Santa Lucia Foundation Istituto di Ricovero e Cura a Carattere Scientifico, Rome, Italy; 2 Data Science Unit, Santa Lucia Foundation Istituto di Ricovero e Cura a Carattere Scientifico, Rome, Italy; 3 Molecular Neuroimmunology Unit, Santa Lucia Foundation Istituto di Ricovero e Cura a Carattere Scientifico, Rome, Italy; 4 Department of Clinical and Behavioral Neurology, Santa Lucia Foundation Istituto di Ricovero e Cura a Carattere Scientifico, Rome, Italy; 5 Medical Services, Santa Lucia Foundation Istituto di Ricovero e Cura a Carattere Scientifico, Rome, Italy; 6 Clinical Microbiology Laboratory, Santa Lucia Foundation Istituto di Ricovero e Cura a Carattere Scientifico, Rome, Italy; 7 Singapore Immunology Network, Agency for Science, Technology and Research, Singapore, Singapore; 8 Genomic Medicine Laboratory Unione Italiana Lotta alla Distrofia Muscolare, Santa Lucia Foundation Istituto di Ricovero e Cura a Carattere Scientifico, Rome, Italy; 9 Medical Genetics Laboratory, Department of Biomedicine and Prevention, Tor Vergata University, Rome, Italy

## Abstract

**Importance:**

The emergence of the highly contagious Omicron variant of SARS-CoV-2 and the findings of a significantly reduced neutralizing potency of sera from individuals with previous SARS-CoV-2 infection or vaccination highlights the importance of studying cellular immunity to estimate the degree of immune protection to the new SARS-CoV-2 variant.

**Objective:**

To determine T-cell reactivity to the Omicron variant in individuals with established (natural and/or vaccine-induced) immunity to SARS-CoV-2.

**Design, Setting, and Participants:**

This was a cohort study conducted between December 20 and 21, 2021, at the Santa Lucia Foundation Istituto di Ricovero e Cura a Carattere Scientifico, Rome, Italy, among health care worker and scientist volunteers. Lymphocytes from freshly drawn blood samples were isolated and immediately tested for reactivity to the spike protein of SARS-CoV-2.

**Main Outcomes and Measures:**

The main outcomes were the measurement of T-cell reactivity to the mutated regions of the spike protein of the Omicron BA.1 SARS-CoV-2 variant and the assessment of remaining T-cell immunity to the spike protein by stimulation with peptide libraries.

**Results:**

A total of 61 volunteers (mean (range) age, 41.62 (21-62) years; 38 women [62%]) with different vaccination and SARS-CoV-2 infection backgrounds were enrolled. The median (range) frequency of CD4^+^ T cells reactive to peptides covering the mutated regions in the Omicron variant was 0.039% (0%-2.356%), a decrease of 64% compared with the frequency of CD4^+^ cells specific for the same regions of the ancestral strain (0.109% [0%-2.376%]). Within CD8^+^ T cells, a median (range) of 0.02% (0%-0.689%) of cells recognized the mutated spike regions, while 0.039% (0%-3.57%) of cells were reactive to the equivalent unmutated regions, a reduction of 49%. However, overall reactivity to the peptide library of the full-length protein was largely maintained (estimated 87%). No significant differences in loss of immune recognition were identified between groups of participants with different vaccination or infection histories.

**Conclusions and Relevance:**

This cohort study of immunized adults in Italy found that despite the mutations in the spike protein, the SARS-CoV-2 Omicron variant was recognized by the cellular component of the immune system. It is reasonable to assume that protection from hospitalization and severe disease will be maintained.

## Key Points

**Question:**

What is the cellular immunity associated with the Omicron variant of SARS-CoV-2 among immunized individuals?

**Findings:**

In this cohort study among 61 individuals who had been vaccinated against COVID-19, cellular responses to the mutated regions of the Omicron spike protein were detected in 80% of participants. The mutations were associated with significantly reduced T-cell recognition compared with the vaccine strain, while reactivity to the whole spike protein was present in 100% of participants, and the proportion of remaining immunity to SARS-CoV-2 was estimated to be 87%.

**Meaning:**

These findings suggest that cellular immunity to the Omicron variant was maintained despite the mutations in its spike protein; thus, immunization may confer protection from severe COVID-19 from the Omicron variant.

## Teaser

This cohort study assesses T-cell reactivity to the Omicron variant of SARS-CoV-2 in individuals vaccinated against SARS-CoV-2.

## Introduction

Far from being weakened, the COVID-19 pandemic has found new strength in another wave of infections with the Omicron variant of SARS-CoV-2.^1^ Mutations in the receptor-binding domain region correlate with lower neutralization potency of sera collected from individuals with immunity from previous infection or vaccination,^2^ setting the stage for immune evasion by the mutated virus. Conveniently, T-cell responses are characterized by vast cross-reactivity,^3^ and cellular immunity is maintained in the face of mutations that may escape antibody recognition.^4^ Still, infection in an immunized individual with a slightly different version of the immunizing pathogen occurs in a context of preexisting immunity, mostly mediated by the cellular component of the immune response. This raises the question of whether the intrinsic cross-reactivity of the spike-specific T cells induced by vaccination or infection will confer a broad enough repertoire for T cells to respond to emerging variants of SARS-CoV-2. Studies on the fine specificity and persistence of spike-specific T cells in individuals with previous SARS-CoV-2 infection have indicated that the cellular response to SARS-CoV-2 offers broad reactivity against spike epitopes,^5,6^ and induction of broadly reactive CD4^+^ and CD8^+^ memory cells has been shown to occur after vaccination as well.^7^ In this study, we investigate the T-cell response to the mutated regions of the spike protein from the Omicron BA.1 variant in 61 individuals who received COVID-19 vaccinations and/or with immunity from previous SARS-CoV-2 infection. By also measuring reactivity to the equivalent regions from the ancestral vaccine strain and to the whole spike protein, we estimate the degree of remaining immunity to the SARS-CoV-2 spike protein.

## Methods

### Study Design and Participants

This cohort study was approved by the ethics committee at the Santa Lucia Foundation Hospital and conducted on December 20 to 21, 2021. All participants provided written informed consent. This study followed the Strengthening the Reporting of Observational Studies in Epidemiology (STROBE) reporting guideline.

Volunteers from among the hospital workers and scientists of the Santa Lucia Foundation donated 15 mL of blood. To include individuals who had been previously infected with SARS-CoV-2, we queried the internal registry to select individuals with positive results on polymerase chain reaction (PCR) tests after periodic surveillance screenings. Participants were divided in 5 groups, based on their vaccination/infection history: (1) those with 2 doses of any 1 vaccine; (2) those with 3 doses of mRNA vaccine; (3) those with heterologous vaccination with adenoviral vector followed by an mRNA vaccine; (4) individuals who had been vaccinated and who had subsequently been infected with SARS-CoV-2; and (5) individuals who had contracted and recovered from SARS-CoV-2 and were subsequently vaccinated (Table).

**Table.** Participant Characteristics

Participant immunological history, No.	Vaccine (in chronological order)	Age, mean (range), y	Sex, men/women
2 Doses (n = 10)			
6	2 BNT162b2	42.5 (23-56)	3/7
3	2 mRNA-1273		
1	2 ChAdOx1-S		
3 Doses of mRNA (n = 15)			
14	3 BNT162b2	52 (26-60)	5/10
1	2 BNT162b2 + 1 mRNA-1273		
Heterologous (n = 11)			
3	2 ChAdOx1-S+ 1 mRNA-1273	53 (26-62)	3/8
6	2 ChAdOx1-S+ 1 BNT162b2		
2	1 Ad26.COV2.S + 1 BNT162b2		
COVID-19 then Vaccine (n = 12)			
7	2 BNT162b2	53.5 (22-60)	8/4
2	3 BNT162b2		
1	1 Ad26.COV2.S		
1	1 mRNA-1273		
1	1 Ad26.COV2.S + 1 mRNA-1273		
Vaccine then COVID-19 (n = 13)			
9	2 BNT162b2	37 (21-54)	4/9
1	1 Ad26.COV2.S		
1	2 ChAdOx1-S+ 1 mRNA-1273		
1^a^	2 ChAdOx1-S		
1^a^	2 BNT162b2		

^a^ Infected with the SARS-CoV-2 Omicron variant.

### T-cell Stimulation

Peripheral blood mononuclear cells were isolated and immediately tested in an in vitro assay, including incubation with 3 different peptide pools: an overlapping peptide pool spanning the entire spike protein from the ancestral vaccine strain (Pool_S_), a pool of 83 peptides covering only the mutated regions of the spike protein from the Omicron variant (Pool_Mut_), and a peptide pool covering the same regions as the mutated Omicron regions but from the ancestral strain (Pool_Ref_) (all pools 1 μg/mL each; Miltenyi Biotec). These peptides pools are 15 mers with 11 amino acid overlap and are ideal for CD4^+^ T-cell stimulation but suboptimal for presentation through major histocompatibility complex Class I, which preferentially binds shorter peptides (8-10 mers), thus CD8^+^ T-cell reactivity may be underestimated. After 18 hours, cells were stained with fluorochrome-conjugated monoclonal antibodies for the detection of the expression of surface activation induced markers (AIM) in CD4^+^ and CD8^+^ T-cell subsets (eTable in the Supplement), and supernatants were collected for measurement of interferon (IFN)-γ release by enzyme-linked immunoassay (Bio-Techne). Activated CD4^+^ cells were defined as activation of CD40 ligand^+^ and CD69^+^ cells, while the expression of CD137^+^ and CD69^+^ identified activated CD8^+^ cells, as previously described.^8^ Following background subtraction of unstimulated cultures, negative values were set to zero. The threshold for positivity was set by calculating the 75th percentile minus the median of the values obtained.^9^ Samples were acquired on Aurora (Cytek) or on Cytoflex LS (Beckman Coulter) flow cytometers. In parallel, 50 μL of corresponding whole-blood samples were stained with anti-CD3, anti-CD4, and anti-CD8 for the determination of absolute cell counts as previously described.^8^ Data were analyzed with FlowJo version 10.8 (BD). Data were visualized using PRISM version 9 (GraphPad).

### Estimation of Remaining Immunity

To estimate the remaining immunity, for each individual we subtracted the number of cells responding to the unmutated pool from the number of cells activated by the complete protein pool to obtain the number of cells specific for the other spike regions. To this, we added the number of cells responding to the mutated pool, corresponding to the cells that had maintained reactivity despite the mutations. Thus, remaining immunity can be estimated with the formula:



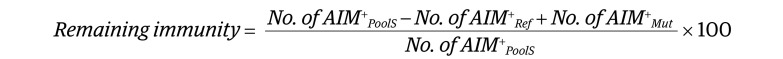



### Statistical Analysis

The differences between groups in CD4^+^ and CD8^+^ activated T cells were assessed for each experimental condition (Pool_S_, Pool_Ref_, and Pool_Mut_) using multiple Kruskal-Wallis rank sum tests. Pairwise post hoc comparisons were performed using the Wilcoxon rank sum test with false discovery rate correction for multiple testing. Within-groups differences in CD4^+^ and CD8^+^ were assessed in Pool_Ref_ and Pool_Mut_ conditions using Friedman rank sum test with Omicron exposure as the fixed effect and participant ID as the random effect. Kendall *W* was used to compute effect size following the Cohen interpretation guidelines (small, 0.1 to <0.3; moderate, 0.3 to <0.5; large, ≥0.5). The obtained effect sizes were used in multiple 2-tailed post hoc power analyses for dependent means to estimate the obtained statistical power with α = .05. The slopes of the regression line between Pool_Ref_ and Pool_Mut_ were computed for CD4^+^ AIM and CD8^+^ AIM for each participant. The obtained slopes were compared between groups using multiple Kruskal-Wallis rank sum tests and Heteroscedastic 1-way analysis of variance for medians with false discovery rate correction. All the statistical analyses were performed using the lme4, lmerTest, and WRS2 libraries in R statistical software version 4.1.2 (R Project for Statistical Computing). Analyses were conducted on December 27, 2021.

## Results

A total of 61 volunteers (mean [range] age, 41.62 [21-62] years; 38 [62%] women) with different vaccination and SARS-CoV-2 infection backgrounds each donated 15 mL of blood, which was immediately processed. Of these participants, 1 had recently completed chemotherapy, and 1 was undergoing treatment with monoclonal antibodies; the others reported no known health issue (Table).

**Figure 1.**
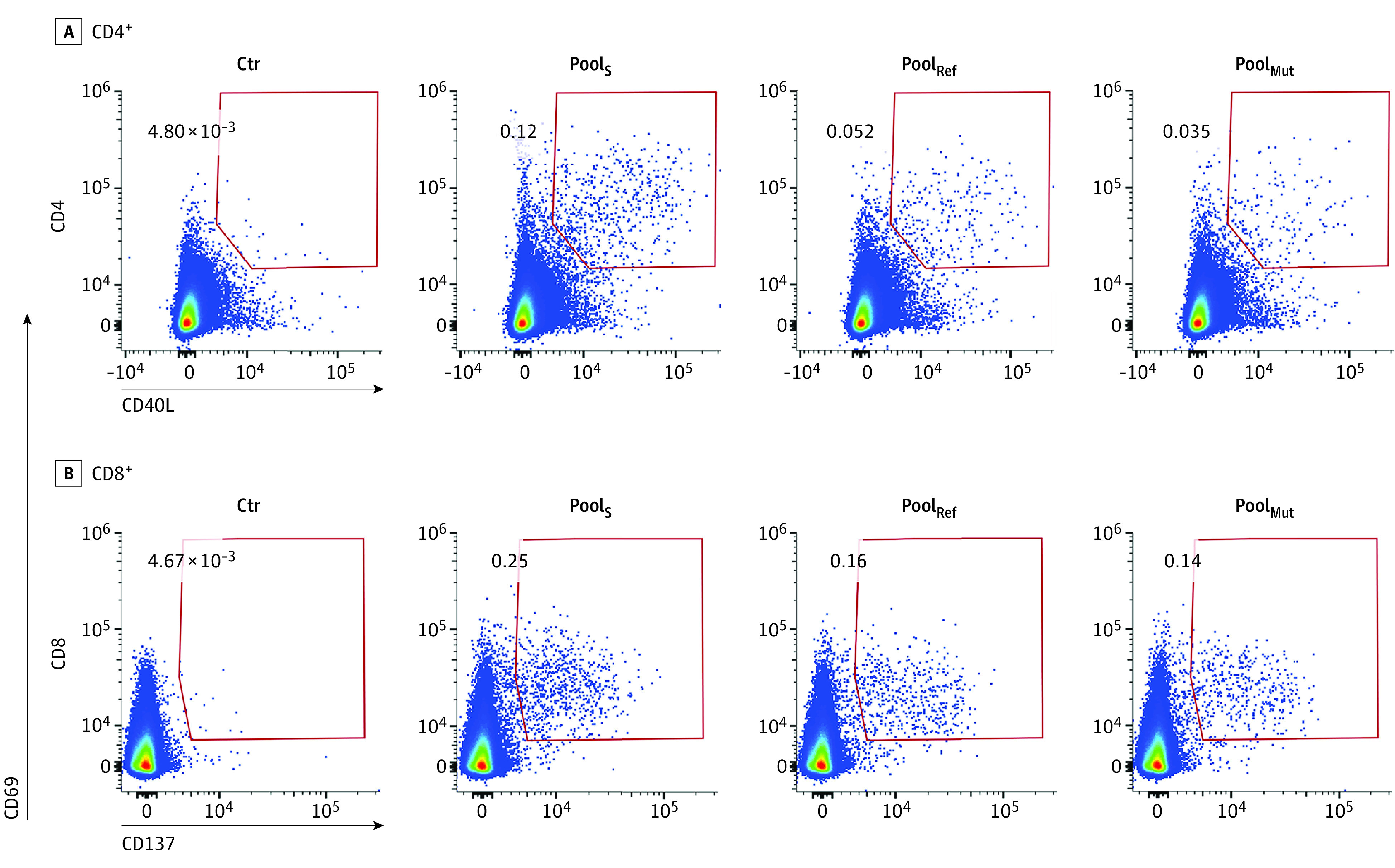
T-cell Responses to the Ancestral and Omicron Spike Protein of SARS-CoV-2 Representative flow cytometry plots gated on CD4^+^ or CD8^+^ T cells showing upregulation of activation markers (CD69 and CD40 ligand [CD40L] for CD4^+^ cells and CD69 and CD137 for CD8^+^ cells) following overnight stimulation with a pool of overlapping peptides covering the whole spike protein from the ancestral vaccine strain (Pool_S_), a peptide pool covering only the mutated regions of the Spike protein from the Omicron variant (Pool_Mut_) or a peptide pool covering the same regions as Pool_Mut_, but from the ancestral strain (Pool_Ref_). Ctr indicates unstimulated control; numbers, percentages of activation induced marker cells within each gate.

**Figure 2.**
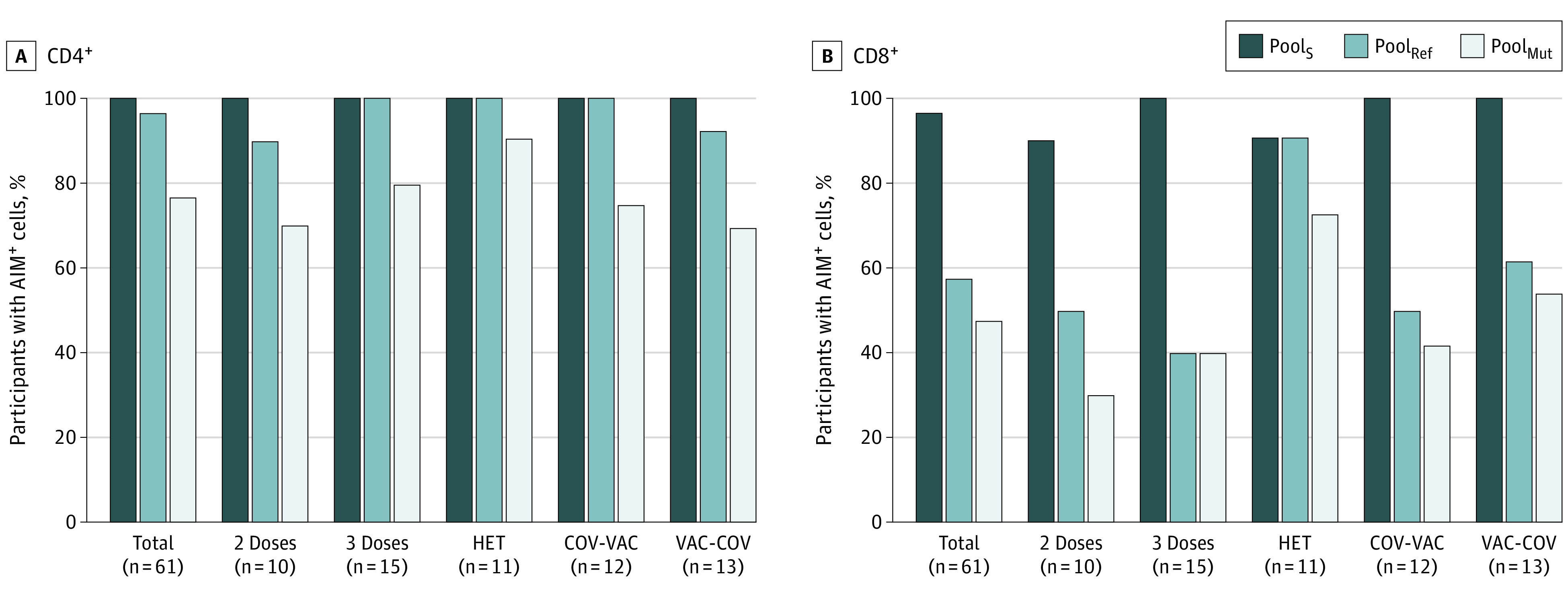
Frequency of Participants in Each Group Presenting Spike-Specific Responses 2 Doses indicates individuals with 2 doses of vaccine; 3 Doses, 3 doses of mRNA vaccine; AIM indicates activation induced marker; COV-VAC, individuals who had contracted and recovered from SARS-CoV-2 and were subsequently vaccinated; HET, heterologous vaccination with adenoviral vector followed by an mRNA vaccine; Pool_Mut_, pool of 83 peptides covering only the mutated regions of the spike protein from the Omicron variant; Pool_Ref_, peptide pool covering the same regions as the mutated Omicron regions, but from the ancestral strain; Pool_S_, an overlapping peptide pool spanning the entire spike protein from the ancestral vaccine strain; and VAC-COV, individuals who had been vaccinated and who had subsequently been infected with SARS-CoV-2.

First, we established T-cell responsiveness to the spike protein (Figure 1). All 61 participants showed CD4^+^ T-cell reactivity to Pool_S_, and 59 participants (97%) showed CD8^+^ T-cell reactivity to Pool_S_ (Figure 2). For Pool_Mut_, CD4^+^ T-cell reactivity was detected in 47 participants (77%), and CD8^+^ T-cell reactivity was detected in 29 participants (48%). For Pool_Ref_, CD4^+^ T cells were present in 59 participants (97%), and CD8^+^ T cells were present in 35 participants (57%). Within the different groups of participants, there was no significant difference in T-cell reactivity to Pool_Mut_ between individuals with the heterologous regimen of vaccination (CD4^+^: 10 of 11 participants [91%]; CD8^+^: 8 of 11 participants [73%]) and those who had received 2 doses of vaccine (CD4^+^: 7 of 10 participants [70%]; CD8^+^: 3 of 10 participants [30%]).

**Figure 3.**
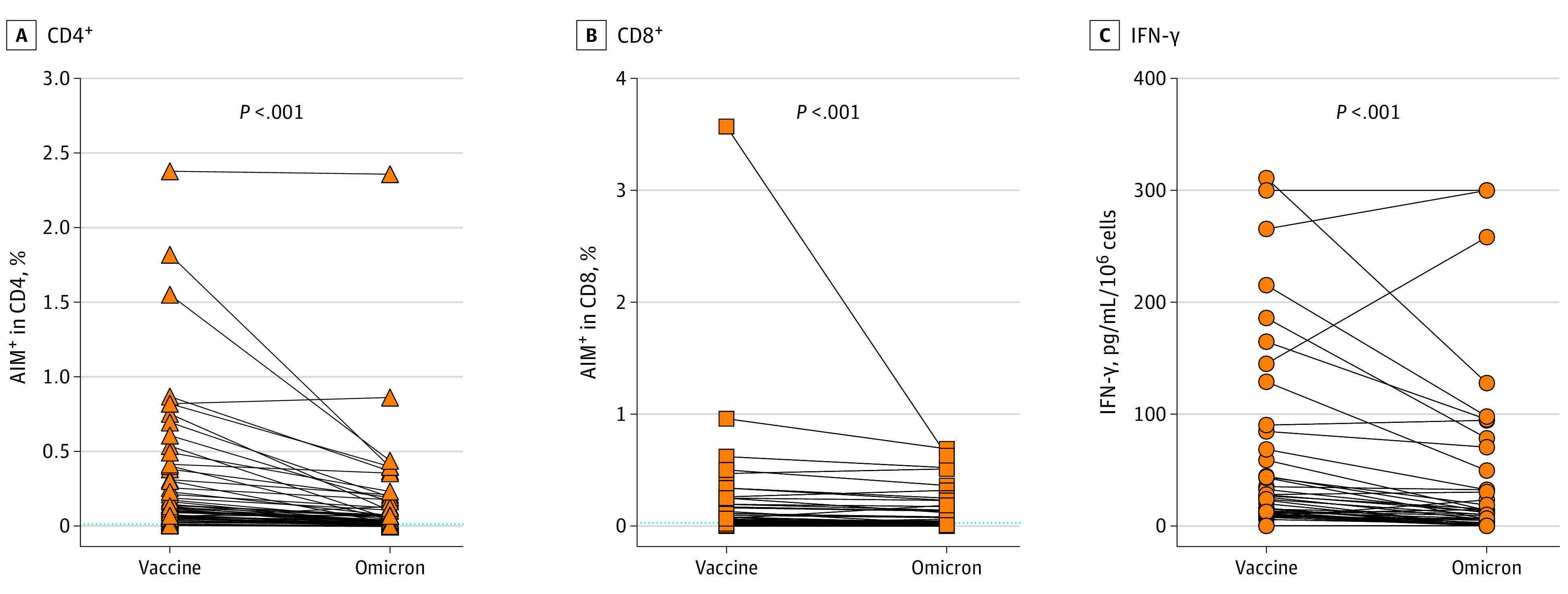
Reduced Recognition of Mutated Regions of the Spike Protein in the Omicron Variant Freshly isolated lymphocytes were incubated with peptide pools encompassing the mutated regions of the spike protein in the Omicron variant (Omicron), and with the reference peptide pool of the same region in the ancestral vaccine strain (Vaccine). Activated CD4^+^ (A) (CD69^+^ and CD40 ligand^+^) and CD8^+^(B) (CD69^+^ and CD137^+^) cells were identified by flow cytometry, and interferon (IFN)-γ production was measured in the supernatants (C). Background T-cell activation in paired unstimulated cultures was subtracted. Dotted lines indicate the threshold for positivity (median − 75th percentile of values from unstimulated cultures). Differences were assessed using Friedman rank sum test with Omicron exposure (vaccine / Omicron) as the random effect and participant ID (points) as the random effect.

Overall, the proportion of T cells activated by Pool_Mut_ was significantly lower than that of T cells recognizing the same regions from the Wuhan strain used for vaccine design (median [range]: CD4^+^, 0.039% [0%-2.356%] vs 0.109% [0%-2.376%]; *P* < .001; CD8^+^, 0.02% [0%-0.689%] vs 0.039% [0%-3.57%]; *P* < .001) (Figure 3). The reduction in T cell numbers reactive to the Omicron variant was found to be significant in all groups of participants, regardless of vaccination and SARS-CoV-2 infection history (eFigure in the Supplement). Individuals who had been infected with SARS-CoV-2 after vaccination showed a lower reduction in the proportion of CD8^+^ T cells recognizing the mutated spike regions. CD8^+^ T-cell reactivity, when present, was more conserved compared with the CD4^+^ subset.

IFN-γ released in the cultures was also significantly reduced when cells were incubated with the peptide pools covering the mutated spike regions, compared with pools spanning the equivalent regions of the ancestral strain (Figure 3; eFigure in the Supplement). Thus, these data show that mutations of the spike protein carried by the Omicron variant were associated with decreased CD4^+^ and CD8^+^ T-cell activation and function.

**Figure 4.**
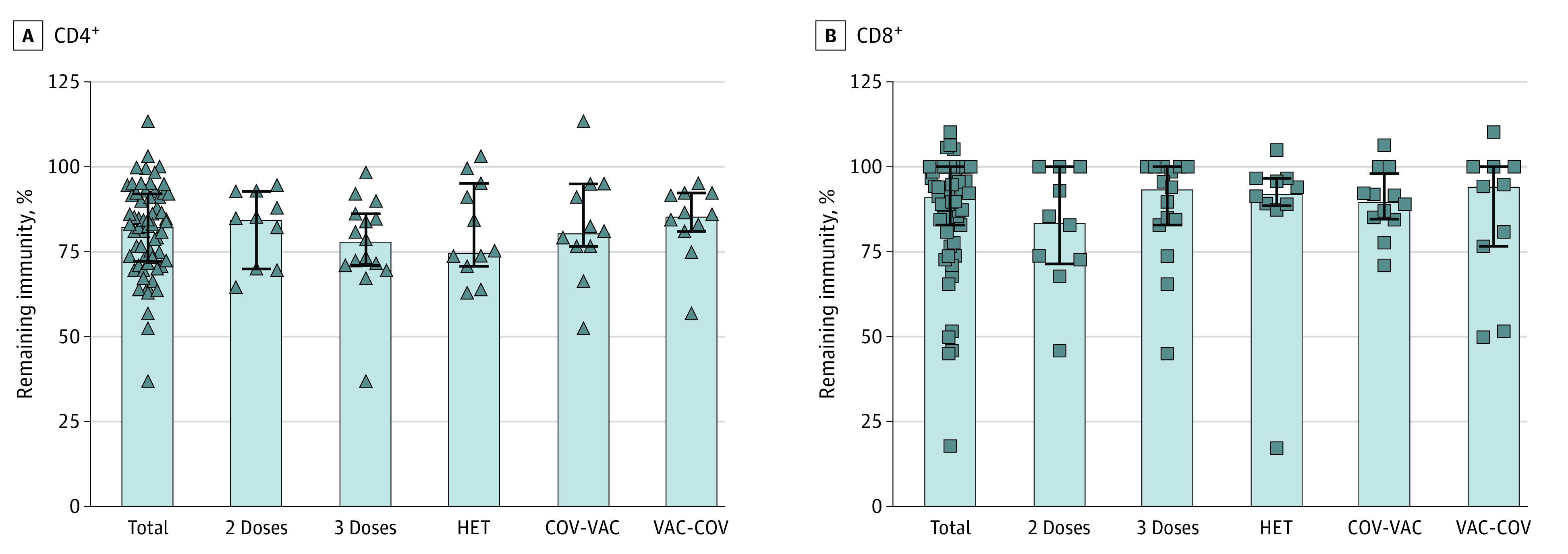
Remaining Immunity to the Spike Protein of the Omicron Variant Fraction of preserved CD4^+^ (A) and CD8^+^ (B) reactivity to the whole spike protein in each group, after having subtracted loss of reactivity to the mutated regions. 2 Doses indicates individuals with 2 doses of vaccine; 3 Doses, 3 doses of mRNA vaccine; COV-VAC, individuals who had contracted and recovered from SARS-CoV-2 and were subsequently vaccinated; HET, heterologous vaccination with adenoviral vector followed by an mRNA vaccine; and VAC-COV, individuals who had been vaccinated and who had subsequently been infected with SARS-CoV-2.

While we observed a significant reduction in T-cell reactivity to the mutated regions of the spike protein from the Omicron variant, these account for a small proportion of the total protein. Using our Equation, we estimate that overall T-cell reactivity to the spike protein of the Omicron variant was maintained by 87% (CD4^+^: 83%; CD8^+^: 91%) (Figure 4), with no significant differences between the different groups of participants.

## Discussion

This cohort study found that that T-cell responses against the mutated regions in Omicron were significantly reduced in immunized individuals. Compared with the Wuhan strain, the Omicron BA.1 variant carries more than 35 mutations in the spike protein. The impact of these mutations on antibody recognition has been shown to be substantial, with a significant loss of neutralizing activity in sera from individuals with immunity from previous infection or vaccination.^2,10^ In previous variants, although antibody neutralizing potency was decreased, T-cell responses were maintained.^4,11^ However, as these regions cover only a small proportion of the whole protein, the overall response against Omicron spike was largely preserved regardless of vaccination and/or infection history in our study, in line with other studies.^12,13,14,15,16^

### Limitations

This study has some limitations. The main limitation is the small number of participants in each group, which resulted in low statistical power for the identification of differences in T-cell responses between groups. Also, we only measured T-cell responses in peripheral blood, which may not fully represent what happens in the respiratory tract and lymph nodes. Additionally, we did not use the whole spike protein from the Omicron variant in our peptide pool, since it was not available at the time of the study. Furthermore, we cannot exclude some distortion due to potential immunodominance effects of the individual peptide epitopes, although no major immunodominance was evident in significantly higher reactivity to the Omicron peptide pool.

## Conclusions

This cohort study of 61 immunized adults in Italy found persisting and robust T-cell responses despite the mutations in the Omicron variant of SARS-CoV-2. These findings suggest that cellular immunity against this variant, together with protection from severe disease, will not be compromised.

## Notes

Supplement. **eFigure.** Reduced Recognition of Mutated Regions of the Spike Protein in the Omicron Variant **eTable.** Flow Cytometry Reagents
