# Supplementary material for: Assessment of T-cell Reactivity to the SARS-CoV-2 Omicron Variant by Immunized Individuals
Source: JAMA Netw Open. 2022 Apr 22;5(4):e2210871. doi: 10.1001/jamanetworkopen.2022.10871 (PMC9034402; doi:10.1001/jamanetworkopen.2022.10871)
Supplement: Supplement. — eFigure. Reduced Recognition of Mutated Regions of the Spike Protein in the Omicron Variant eTable. Flow Cytometry Reagents [file jamanetwopen-e2210871-s001.pdf]

Supplemental Online Content

De Marco L, D’Orso S, Pirronello M, et al. Assessment of T-cell reactivity to the SARS-CoV-2 Omicron variant by immunized individuals. *JAMA Netw Open*. 2022;5(4):e2210871. doi:10.1001/jamanetworkopen.2022.10871

eFigure. Reduced Recognition of Mutated Regions of the Spike Protein in the Omicron Variant
eTable. Flow Cytometry Reagents

This supplemental material has been provided by the authors to give readers additional information about their work.

© 2022 De Marco L et al. *JAMA Network Open.*

eFigure. Reduced Recognition of Mutated Regions of the Spike Protein in the Omicron Variant

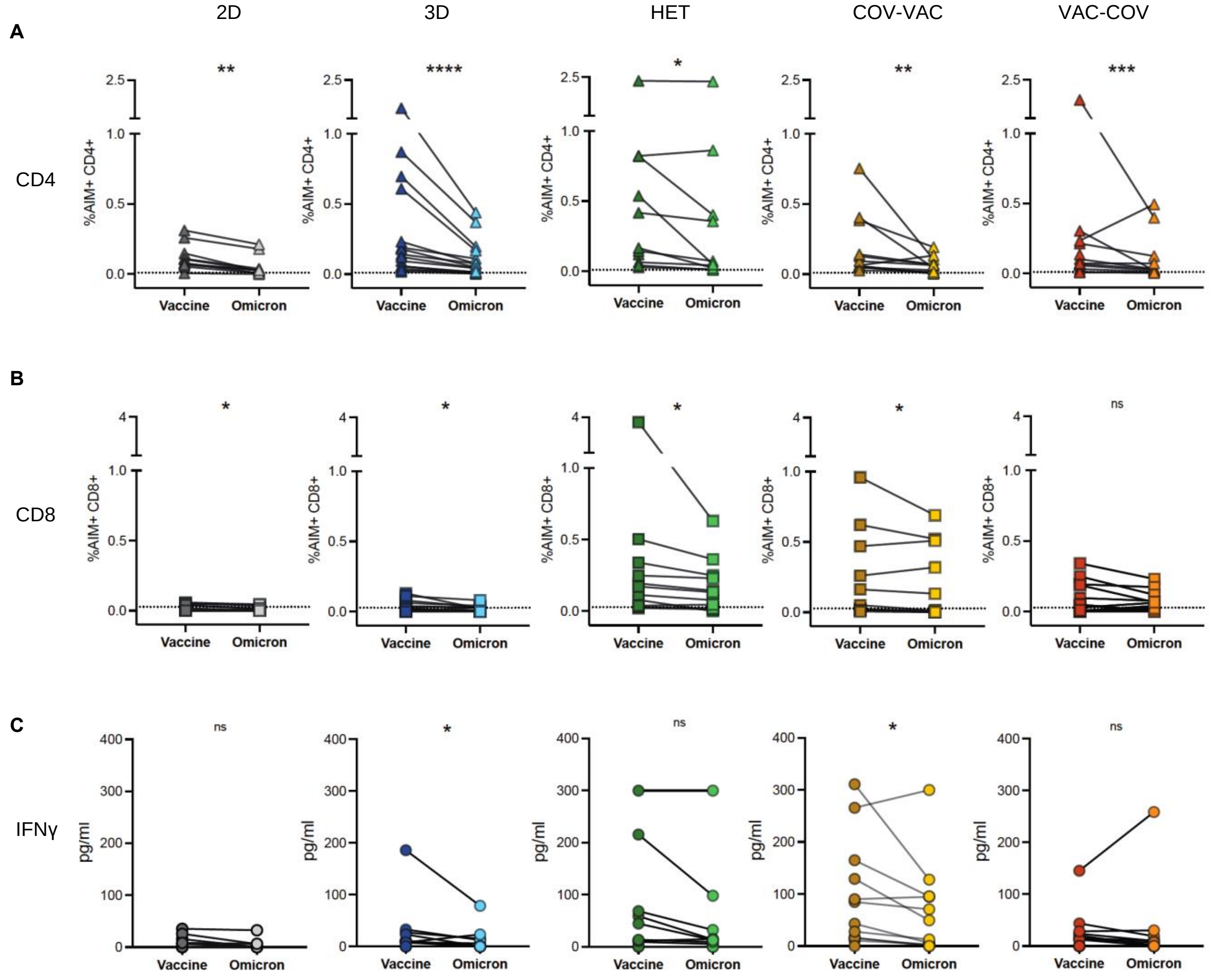

Freshly isolated lymphocytes were incubated with peptide pools encompassing the mutated regions of the Spike protein in the Omicron variant (Omicron), and with the reference peptide pool of the same region in the ancestral vaccine strain (Vaccine). Activated CD4+ (A) (CD69+CD40L+) and CD8+(B) (CD69+CD137+) cells were identified by flow cytometry, and IFN-γ production was measured in the supernatants (C). Background T cell activation in paired unstimulated cultures was subtracted. Dotted lines indicate the threshold for positivity (median- 75th percentile of values from unstimulated cultures). 2D: = 2 doses of vaccine; 3D: 3 doses mRNA vaccine; HET: 1-2 doses adenoviral vector vaccine + 1 dose mRNA; COV-VAC: covid convalescents subsequently vaccinated; VAC-COV Vaccinated individuals subsequently infected with SARS-CoV-2. Differences were assessed using Friedman rank sum test with omicron exposure (vaccine/omicron) and subject ID as fixed and random effects respectively. *P < 0.05; **P < 0.01; ***P < 0.001; ****P < 0.0001; N.S. Not Significant

© 2022 De Marco L et al. *JAMA Network Open.*

eTable. Flow Cytometry Reagents

Antibody	Fluorochrome	Clone	Company	Titer
CD14	FITC	REA599	Miltenyi	1:100
CD19	FITC	MAB-1118F	ImmunolScience	1:30
CD69	BB700	FN50	Bect.Dick.	1:100
CD154	PE	24-31	eBioscience	1:30
CD4	PECy5.5	13B8.2	Coulter	1:100
CD8	BV605	SK1	Bect. Dick.	1:40
CD137	BUV395	4B4-1	Bect. Dick.	1:30
CD3	BUV496	UCHT1	Bect.Dick.	1:50
LD	Near-IR		ThermoFisher	1:60

© 2022 De Marco L et al. *JAMA Network Open.*
